# Interaction Effects of Ultrafine Carbon Black with Iron and Nickel on Heart Rate Variability in Spontaneously Hypertensive Rats

**DOI:** 10.1289/ehp.9821

**Published:** 2007-02-27

**Authors:** Chuen-Chau Chang, Jing-Shiang Hwang, Chang-Chuan Chan, Tsun-Jen Cheng

**Affiliations:** 1 Institute of Occupational Medicine and Industrial Hygiene, National Taiwan University, Taipei, Taiwan; 2 Department of Anesthesiology, Taipei Medical University Hospital, Taipei, Taiwan; 3 Institute of Statistical Science, Academia Sinica, Taipei, Taiwan

**Keywords:** ambient particles, heart rate variability, interaction, iron, nickel, spontaneously hypertensive rats, ultrafine carbon black

## Abstract

**Background:**

Particulate matter (PM) has been reported to be associated with alterations in heart rate variability (HRV); however, the results are inconsistent. We propose that different components of PM cause the discrepancy.

**Objective:**

In this study, our goal was to determine whether different types of exposure would cause different HRV effects, and to verify the interactions between co-exposing components.

**Methods:**

Ultrafine carbon black (ufCB; 14 nm; 415 μg and 830 μg), ferric sulfate [Fe_2_(SO_4_)_3_; 105 μg and 210 μg], nickel sulfate (NiSO_4_; 263 μg and 526 μg), and a combination of high-dose ufCB and low-dose Fe_2_(SO_4_)_3_ or NiSO_4_ were intratracheally instilled into spontaneously hypertensive rats. Radiotelemetry data were collected in rats for 72 hr at baseline and for 72 hr the following week to determine the response to exposure. Effects of exposure on 5-min average of normal-to-normal intervals (ANN), natural logarithm-transformed standard deviation of the normal-to-normal intervals (LnSDNN), and root mean square of successive differences of adjacent normal-to-normal intervals (LnRMSSD) were analyzed using self-control experimental designs.

**Results:**

Both high- and low-dose ufCB decreased ANN marginally around hour 30, with concurrent increases of LnSDNN. LnRMSSD returned to baseline levels after small initial increases. We observed minor effects after low-dose Fe and Ni instillation, whereas biphasic changes were noted after high-dose instillations. Combined exposures of ufCB and either Fe or Ni resulted in HRV trends different from values estimated from individual-component effects.

**Conclusions:**

Components in PM may induce different cardioregulatory responses, and a single component may induce different responses during different phases. Concurrent exposure to ufCB and Fe or Ni might introduce interactions on cardioregulatory effects. Also, the effect of PM may be mediated through complex interaction between different components of PM.

Recently, cardiovascular morbidity and mortality have been linked to particulate matter (PM) air pollution (Lanki et al. 2006; Pekkanen et al. 2002; Samet et al. 2000; Zanobetti and Schwartz 2005) and ranked as one of the most costly types of PM-related death (Dockery 2001). The PM-associated activation of the autonomic nervous system, usually expressed as changes in heart rate variability (HRV), has been postulated as one of the major mechanisms linking PM exposures and their cardiovascular effects in the most recent studies (Brook et al. 2004).

Epidemiologic studies (Chuang et al. 2005b; Samet et al. 2000; Schwartz et al. 1999; Seaton et al. 1999), especially those focusing on HRV indices (Chan et al. 2004, 2005; Chuang et al. 2005a; Gong et al. 2004; Tarkiainen et al. 2003; Timonen et al. 2006; Wheeler et al. 2006), have demonstrated that PM-mediated cardiovascular effects are heterogeneous, depending on particle contents. Animal models have been used to investigate the effects of different particles (Campen et al. 2001, 2002; Chen et al. 1992; Gordon et al. 1998; Kodavanti et al. 1998, 2002; Ulrich et al. 2002). The cardiovascular toxicities were also demonstrated to be heterogeneous in toxicologic settings focusing on cytokine release (Hetland et al. 2005; Li et al. 1999), heart rate changes (Gordon et al. 1998; Wellenius et al. 2003), electrocardiographic (ECG) changes (Gordon et al. 2000; Hwang et al. 2005; Wellenius et al. 2003), and HRV indices (Chen and Hwang 2005; Godleski et al. 2000). All of these toxicologic studies support the heterogeneity of PM cardiovascular effects. However, the underlying mechanisms remain to be explored.

Several studies have been devoted to determining the explanation for these observed heterogenic PM effects, including the vulnerability and host effects (Bateson and Schwartz 2004; Schwartz et al. 2005). It has also been postulated that the compositional characteristics of particles may contribute to their different health impacts (Ostro et al. 2007). This hypothesis is supported both by epidemiologic observations, and animal toxicologic research. The most appealing observations include the association between HRV indices and trajectory (Godleski et al. 2000; Lippmann et al. 2006), and componential groups by statistical modeling (Lippmann et al. 2005). These observations suggested that the cardiovascular effects of PMs varied significantly with their compositional characteristics. Our objectives in this study are to verify that different PM components can cause different cardioregulatory effects; that a single PM component exposure can cause different effects at different phases; and that combined exposure to multiple PM components can produce interactions modulating the final outcomes.

## Materials and Methods

### Experimental design

We obtained 60-day-old male spontaneously hypertensive (SH) rats from the National Laboratory Animal Breeding and Research Center (Taipei, Taiwan). They were housed individually on Aspen chip bedding and provided with Lab Diet 5001 (PMI Nutrition International, Richmond, IN, USA) and water *ad libitum*. A 12-hr light/dark cycle, a constant room temperature, and a constant relative humidity were maintained in the animal room during the study.

SH rats were implanted with radio-telemetry transmitters at 11 weeks of age. Experiments were performed over a 2-week period, beginning 10 days after implantation. Phosphate buffered saline (PBS) was given via intratracheal (IT) instillation (under Sevoflurane general anesthesia; Abbott Laboratories Ltd., Queenborough, Kent, UK) at a volume of 0.25 mL per animal in the first week. Radiotelemetric data were subsequently collected for 72 hr, and this served as a baseline template in the analysis process. Materials to be tested were suspended or dissolved in PBS, and were given to the same animals on the same day and time the following week. Data collected for the next 72 hr served as the response data.

**Figure 1:**
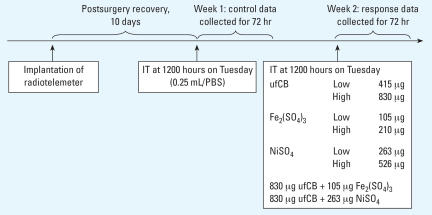
Summary of IT instillation protocol. See “Materials and Methods” for details of experiments. Data collected from the first week (PBS alone) served as controls for data from the second week (test materials).

As shown in Figure 1, eight groups of experiments were performed. Test materials were dispersed or dissolved in 0.25 mL PBS: 14 nm ultrafine carbon black (ufCB; low-dose: 415 μg/animal, *n* = 4; and high-dose: 830 μg/animal, *n* = 4); ferric sulfate [Fe_2_(SO_4_)_3_; low-dose: 105 μg/animal, *n* = 5; high-dose: 210 μg/animal, *n* = 3]; and nickel sulfate (NiSO_4_; low-dose: 263 μg/animal, *n* = 5; high-dose: 526 μg/animal, *n* = 3). Concomitant exposures of 830 μg ufCB with 105 μg Fe_2_(SO_4_)_3_ (*n* = 4) or 263 μg NiSO_4_ (*n* = 4) in 0.25 mL PBS were also performed. All materials to be tested underwent ultrasonication for 30 min before IT instillation. The exposure dosages of ufCB were previously determined in our laboratory. The low exposure dosages of Fe_2_(SO_4_)_3_ and NiSO_4_ were comparable with those used in previous works (Campen et al. 2002); we then doubled those doses to investigate more significant HRV changes. All protocols used in this study were approved by the Committees on Use and Care of Animals of the National Taiwan University. All SH rats used in this study were treated humanely according to institutional guidelines, with appropriate consideration for the alleviation of suffering and distress.

### Heart rate variability measurements

The methodology of HRV measurements with the radiotelemetry system has been described previously (Chang et al. 2004, 2005; Cheng et al. 2003). Briefly, we collected all ECG signals throughout the study on a continuous basis. The sampling rates for the ECG signals were set at 1,000 points per second (1,000 Hz) for better temporal discrimination.

Time intervals between adjacent R waves in the ECG recording (RR) were calculated on a beat-to-beat basis using Dataquest A.R.T. Analysis software, version 2.20 (Data Sciences International, St. Paul, MN, USA). To obtain normal-to-normal (NN) intervals, we used a computer algorithm based on the recommendation by Cheung (1981) to eliminate type A and type B errors in NN calculation. Basically, the NN calculation followed a two-step procedure: the increase or decrease of any RR compared with the previous RR was limited to 33% in a step-1 correction, and data points with distances to the median > 1.5 SDs on Lorenz plots were eliminated in step 2 for every 30 min. The 5-min SD of the normal-to-normal intervals (SDNN) and the root mean square of successive differences of adjacent normal-to-normal intervals (RMSSD) were then calculated from these NN data sets.

### Statistical analysis

Owing to individual variation among diseased animals, conventional exposure–control experimental designs would necessitate a large sample size to demonstrate minute effects under strong confounding conditions, a scenario commonly seen in the study of PM toxicology. Furthermore, the circadian nature of cardiovascular parameters often complicates the analysis. Thus, we used a self-control experimental designs in this research. Exposures were carried out at the same time (starting from 1200 hours) on Tuesday of two consecutive weeks. Data collected in the first week (animals exposed to PBS alone) served as the control group for those collected in the following week (animals exposed to test materials).

**Figure 2:**
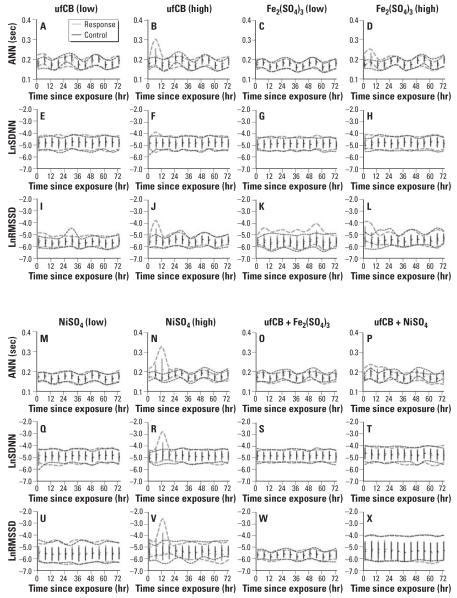
Original data distribution of response and baseline (control) for ANN, LnSDNN, and LnRMSSD for low-dose ufCB (*A, E*, and *I*), high-dose ufCB (*B, F*, and *J*), low-dose Fe_2_(SO_4_)_3_ (*C, G*, and *K*), high-dose (*D, G*, and *L*), low-dose NiSO_4_ (*M, Q*, and *U*), high-dose NiSO_4_ (*N, R*, and *V*), ufCB + Fe_2_(SO_4_)_3_ (*O, S*, and *W*), and ufCB + NiSO_4_ (*P, T*, and *X*). Values shown are mean ± SE; dashed lines represent the 95% distribution envelopes (the region of distribution for 2.5 percentile up to 97.5 percentile data points).

We calculated the SDNN and RMSSD as described previously (Chang et al. 2005). Average NN intervals (ANN) and natural logarithm transformation of SDNN (LnSDNN) and RMSSD (LnRMSSD) were used as outcome measurements to produce approximately symmetrical distributions of response variables for statistical analysis. Time plots of the original data are shown in Figure 2.

We calculated hourly means of the control group data of all three HRV parameters (ANN, LnSDNN, and LnRMSSD); these hourly means served as circadian templates in the analytic procedures. To better illustrate particle effects, we subtracted the hourly means from all 5-min data from each HRV parameter to obtain crude effects, which were then used for the computation of 6-hr average crude effects.

We used the generalized estimation equation (GEE) model to further examine the exposure effect during the 72-hr observation period. We modeled the exposure effects with a set of 13 dummy variables, each standing for average crude effects of the 1-hr preparation and 6-hr succeeding time segments. *Y_it_* is the average crude effect for the *i*th SH rat at time *t* = 0, 1, 2, … 12, in which *t* = 0 corresponds to the 1-hr preparation, and the following time points correspond to the 12 6-hr sections during the 72-hr observation period. For adjusting rat-to-rat variation and control group effects, dummy variables for the number (*n*) of animals and the 13 HRV parameter values *B_t_* obtained in the first week are included in the model. Specifically, the GEE model is given by


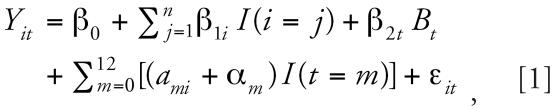


where *i* = 1, …, *n*, *t* = 0, 1, 2, … 12, and *I* (·) is an indicator function. We chose the error term ɛ*_it_* to be an autoregressive process with order 1 to model time dependence. The coefficients α*_m_* , for *m* = 0, 1, 2, … 12, were used to describe the 6-hr mean exposure effects during the 72-hr observation period. Because the SH rats were randomly selected from a population, in addition to the overall difference parameter β_1_*_i_* , we added random components *a_mi_* to model the rat-to-rat variation of these effects. All of these random coefficients were assumed to be normally distributed with the mean of 0 and some constant variances. The time plots of the estimation of 6-hr means and 95% confidence intervals (CIs) were generated to provide an overall impression of the data.

The exposure effects of high-dose ufCB and low-dose Fe_2_(SO_4_)_3_ or NiSO_4_ were used to generate two virtual series by temporal summation of means and variances of the data from every 6 hr. These two virtual series were defined as the expected combined effects. Time plots of real combined effects of high dose ufCB and low dose Fe_2_(SO_4_)_3_ or NiSO_4_ were used to analyze the interactions between ufCB and Fe_2_(SO_4_)_3_ or NiSO_4_.

We used SAS 8.2 statistical software package (SAS Institute, Cary, NC, USA) to manage data and estimate the parameters and standard errors in the models.

## Results

### Exposure effects

**Figure 3:**
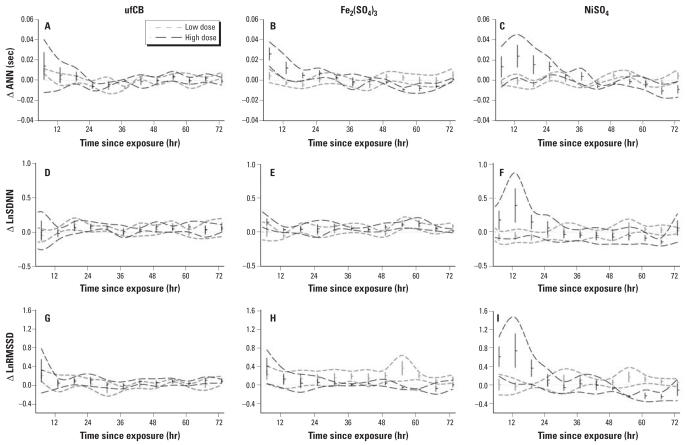
Dosage effects and dynamic responses for low-dose and high-dose ufCB (*A, D*, *G*), Fe_2_(SO_4_)_3_ (*B, E*, *H*), and NiSO_4_ (*C, F*, *I*). (*A–C*) ΔANN. (*D–F*) ΔLnSDNN. (*G–I*) ΔLnRMSSD. Values shown are mean ± SE; dashed lines indicate 95% CI.

The GEE model–estimated exposure effects of ufCB, Fe_2_(SO_4_)_3_, and NiSO_4_ are shown in Figure 3. As shown in Figure 3, for both low-dose (415 μg) and high-dose (830 μg) ufCB exposures, the ANN basically exhibited a borderline depressed level centering around 30 hr after exposure (Figure 3A). Increased LnSDNN was followed by nonsignificant changes (Figure 3D). Initially elevated LnRMSSD followed a back-to-baseline trend 6 hr after exposure (Figure 3G).

Exposure to low-dose (105 μg) Fe_2_(SO_4_)_3_ resulted in increased LnSDNN at the end of the 72-hr observation (Figure 3E). The increase in LnRMSSD was small and persistent, and reached significance in the latter half of the observation period (Figure 3H). Exposure to high-dose (210 μg) Fe_2_(SO_4_)_3_ resulted in significantly biphasic responses in ANN (Figure 3B) and LnRMSSD (Figure 3H), which increased in the first 24 hr and decreased in the last 24 hr. Increased LnSDNN in the first and last 24 hr, however, rendered the responses multimodal (Figure 3E).

The exposure effects of NiSO_4_ are shown in Figure 3C, 3F, and 3I. Exposure to low-dose (263 μg) NiSO_4_ did not generate prominent responses in HRV measurements. In contrast, exposure to high-dose (516 μg) NiSO_4_ resulted in biphasic responses in all three measurements, which increased in the first 24 hr and decreased in the last 24 hr.

### Expected and real combined effects

**Figure 4:**
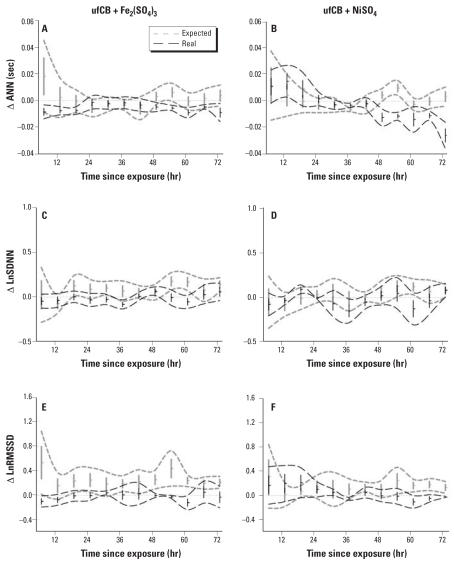
Interactions between ufCB and transition metals shown as expected and real combined effects. ΔANN (*A, B*), ΔLnSDNN (*C, D*), and ΔLnRMSSD (*E–F*) for ufCB + Fe_2_(SO_4_)_3_ (*A, C, E*), and ufCB + NiSO_4_ (*B, D*, *F*). Values shown are mean ± SE; dashed lines represent 95% CIs.

The expected and real combined effects are illustrated in Figure 4. The expected combined effects in both Fe_2_(SO_4_)_3_ and NiSO_4_ basically followed similar trends. Compared with the expected combined effects, the real combined effects of ufCB and Fe _2_ (SO _4_ ) _3_ tend to demonstrate milder changes in all three parameters during the 36 hr after exposure. In the combined exposure of ufCB and NiSO_4_, the real combined effects had a tendency to show more prominent changes in all three parameters during the same period. For the last 24 hr, the real combined effects for both groups tracked comparable trends, and were frequently separated from the expected combined effects.

## Discussion

### Dose responses to ufCB

Effects of high-dose ufCB were not obviously different from those of low-dose ufCB. According to Harder et al. (2005), the decrease in ANN around 30 hr after ufCB instillation might reflect low-grade but significant pulmonary inflammation. Failure of high-dose ufCB to induce more prominent responses might be due to aggregation effects. Although ultrasonication was applied to all materials before instillation, the aggregation of ufCB still should be considered, particularly when the concentrations are high (Gilmour et al. 2004).

### Dose and phased responses to Fe_2_(SO_4_)_3_ or NiSO_4_

Whereas reactions to low-dose Fe_2_(SO_4_)_3_ and NiSO_4_ were modest, the responses to high doses were noticeably biphasic in the present study. Campen et al. (2002) found that low-dose Fe_2_(SO_4_)_3_ produced no obvious changes in heart rate and core body temperature on monocrotaline-induced pulmonary hypertensive Sprague-Dawley rats, whereas NiSO_4_ demonstrated acute bradycardia. In the present study, we found increased LnSDNN and LnRMSSD toward the end of the 72-hr observation period in response to the same level of Fe_2_(SO_4_)_3_. Exposure to low-dose NiSO_4_ did not result in significant HRV changes. We thus tested the HRV responses to “double-dosed” exposures, and demonstrated biphasic effects. The difference of the effects between these studies might have derived from model dissimilarity (Chang et al. 2004; Cheng et al. 2003).

Decreased ANN and LnSDNN 48 hr after IT instillation of Ni are comparable with the most recent study found on ApoE^–/–^ (apolipoprotein deficient) mice by inhalation of Ni-rich concentrated ambient particles (Lippmann et al. 2006). Biological plausibility of Ni-induced cardiovascular effects was well reviewed in their work and is applicable to our results. The discrepancy between response phases may be caused by differences in the model and the experimental design.

### Significance and possible mechanisms of phased responses

Single-phased and dose-dependent decreases in heart rate have been demonstrated in SH rats after IT instillation of an oil combustion–derived PM rich in transition metals (Wichers et al. 2004). Although biphasic heart rate and thermoregulatory effects have been demonstrated in cardiopulmonary-compromised rats exposed to residual oil fly ash (Campen et al. 2000; Watkinson et al. 1998), this is not the case in single component exposures. Conversely, exposure to high-dose Fe_2_(SO_4_)_3_ and NiSO_4_ generated biphasic changes in all three parameters in the present study. To the best of our knowledge, this is the first study to demonstrate biphasic HRV responses to single component exposures.

We have speculated that the time lag and complex interplay among incoming C-fiber stimulation, reactive oxygen species (ROS) production (Adler et al. 1999; Avshalumov et al. 2000; Girouard and de Champlain 2005; Zanzinger and Czachurski 2000), and inflammation with proinflammatory cytokines release (Elder et al. 2004; Hirano et al. 1994; Kang et al. 2002; Lei et al. 2004a, 2004b, 2005; Shwe et al. 2005; Tracey 2002; Yang et al. 1997) might all contribute to the synthesis of the observed biphasic responses. However, this speculation warrants further testing and verification.

### Interactions between ufCB and/or Fe_2_(SO_4_)_3_ or NiSO_4_

The expected combined effects are the virtual series generated by temporal summation of means and variances of the real exposure effects of high-dose ufCB and low-dose Fe_2_(SO_4_)_3_ or NiSO_4_. Because the aim of the present study was to examine interactions between ufCB and transition metals, we selected transition metals at doses that produced minimal HRV effects: low-dose Fe_2_(SO_4_)_3_ and NiSO_4_. Because neither low nor high doses of ufCB generate significantly different HRV effects, we chose high-dose ufCB for more complete “absorption” of transition metals on the carbonaceous surfaces. In the present study, combined exposures of ufCB and Fe_2_(SO_4_)_3_ or NiSO_4_ demonstrated real combined effects that were significantly different from the expected combined effects. These trends verified significant interactions between the exposure components.

Transition metals have demonstrated interactions on cardioregulatory and thermo-regulatory effects (Campen et al. 2002). Interactions between ufCB and Fe were also verified on pulmonary inflammation and ROS production (Wilson et al. 2002). We speculate that these interactions might involve a complex interplay among chelating/leaching kinetics, inflammatory processes, and ROS reactions (Arimoto et al. 2005). Ambient ufCB and transition metals provoke different cardioregulatory effects when administered jointly, and these effects might be augmented in compromised vulnerable subjects. This speculation deserves further research and verification.

### Experimental niches and limitations

#### IT under general anesthesia

To precisely control the dosage, we used IT instillation as the exposure route. However, this procedure is considered invasive and less physiologic (Driscoll et al. 2000) and requires general anesthesia. We chose the ultra-short inhalation anesthetic Sevoflurane to shorten the postanesthetic recovery to within 2 min, and we discarded data acquired within the first hour. This new and improved technology has minimized the anesthesia-associated variations to a negligible level. IT instillation disperses the particles evenly throughout most airways independent of particle size (Leong et al. 1998). We believe that, within the lung, the pattern of distribution of instilled ufCB, Ni, and Fe compounds is similar, and that the response discrepancy might not have originated from distribution pattern differences.

#### SH rats

Kodavanti et al. (2000) and Watkinson et al. (2001) observed exacerbated cardiopulmonary injury and oxidative stress in SH rats exposed to PM and concluded that the SH rat is a potentially useful model to study the susceptibility to PM effects on the cardiovascular system. In the present study we used SH rats as an oxidation-deficient animal model, and we suggest that this model might be useful in assessing the potential biological plausibility linking PM exposures and the cardioregulatory effects in subpopulations with increased oxidative stress (Schwartz et al. 2005). Although SH rats have been suggested to be suitable for mimicking human essential hypertensive subgroups (Sun and Zhang 2005), their pathophysiology may not completely match that of humans (Watkinson et al. 2003). Extrapolation of these PM-associated cardiovascular effects to human beings deserves further studies on healthy controls, including Wistar-Kyoto rats.

#### Time domain HRV

In the present study, only time domain HRV parameters were used to measure the cardioregulatory effects, sparing the more sophisticated frequency domain parameters. However, we have previously demonstrated the applicability of these parameters (Chang et al. 2005) and the correlation with other hemodynamic indices (Chang et al. 2004). Owing to the close correlations among these parameters and those of frequency domain (Kleiger et al. 1991), this limitation does not seriously restrict the interpretation of results. Besides, the index ANN is equivalent to the inverse of heart rate in beats per minute. The use of ANN may cause some inconvenience in biologic interpretation, but it complies with HRV analysis for better symmetry of data distribution.

#### Statistical strategy and experimental design

Owing to technical demands, large-scaled experiments were impractical in our study. We used self-control experimental designs and GEE models to remedy the interference introduced by the relatively small sample size. In contrast, only three PM components were used in the present study, sparing many others. These factors have limited the scope of the study to some extent. However, to the best of our knowledge, this is the first study investigating the interactions between ufCB and transition metals on HRV and might indicate further investigations on many other major PM components for their dynamic effects and potential interactions.

#### Concomitant exposure to ufCB and transition metals

In the present study, we presume that administering Ni or Fe individually is comparable to having these two substance leach from ufCB once instilled. A recent study investigating the interaction between 14 nm ufCB and transition metals on pulmonary inflammation and ROS formation also used a similar approach (Wilson et al. 2002). However, the kinetics of Ni or Fe leaching from ufCB has not been completely studied. We suggest that caution is required in interpretation before more detailed binding/leaching kinetics are available.

## Conclusion

In the present study, we demonstrated that ufCB has different cardioregulatory effects at different phases. The HRV responses to high-dose Fe_2_(SO_4_)_3_ and NiSO_4_ were noticeably biphasic, although the reactions to low-dose exposures were modest. Whereas the dose effects of Fe_2_(SO_4_)_3_ and NiSO_4_ were obvious, those for ufCB were obscure. Concurrent exposure to ufCB and Fe_2_(SO_4_)_3_ or NiSO_4_ introduced cardioregulatory responses that were more significant than those to single-component exposures.

We concluded that different components in PM might induce different cardioregulatory effects. A single-component exposure might also induce different effects at different phases, resulting in biphasic or even more complex cardioregulatory responses. Combined exposure to multiple components could introduce interactions among copollutants, and temporal summation of componential toxic responses might not be appropriate in the estimation of cardiovascular effects in real-life exposures.
